# Salvage peptide receptor radionuclide therapy with [^177^Lu-DOTA,Tyr^3^]octreotate in patients with bronchial and gastroenteropancreatic neuroendocrine tumours

**DOI:** 10.1007/s00259-018-4158-1

**Published:** 2018-09-28

**Authors:** W. A. van der Zwan, T. Brabander, B. L. R. Kam, J. J. M. Teunissen, R. A. Feelders, J. Hofland, E. P. Krenning, W. W. de Herder

**Affiliations:** 1 Department of Radiology & Nuclear Medicine, Erasmus Medical Centre, Rotterdam, The Netherlands; 2 Department of Internal Medicine, Erasmus Medical Centre, Rotterdam, The Netherlands; 3 Cyclotron Rotterdam BV, Erasmus Medical Centre, Rotterdam, The Netherlands

**Keywords:** PRRT, Salvage, Efficacy, Safety, Gastroenteropancreatic, Neuroendocrine

## Abstract

**Purpose:**

Therapy with [^177^Lu-DOTA,Tyr^3^]octreotate is effective in patients with grade I/II metastasized and/or inoperable bronchial neuroendocrine tumour (NET) or gastroenteropancreatic NET (GEP-NET). In this study, we investigated the efficacy and safety of salvage treatment with [^177^Lu-DOTA,Tyr^3^]octreotate.

**Methods:**

Patients with progressive bronchial NET or GEP-NET were selected for re-(re)treatment if they had benefited from initial peptide receptor radionuclide therapy (I-PRRT) with a minimal progression-free survival (PFS) of 18 months. Patients received an additional cumulative dose of 14.8 GBq of [^177^Lu-DOTA,Tyr^3^]octreotate over two cycles per retreatment with PRRT (R-PRRT) or re-retreatment with PRRT (RR-PRRT).

**Results:**

The safety and efficacy analyses included 181 patients and 168 patients, respectively, with bronchial NET or GEP-NET. Overall median follow-up was 88.6 months (95% CI 79.0–98.2). Median cumulative doses were 44.7 GBq (range 26.3–46.4 GBq) during R-PRRT (168 patients) and 59.7 GBq (range 55.2–≤60.5 GBq) during RR-PRRT (13 patients). Objective response and stable disease, as best response, were observed in 26 patients (15.5%) and 100 patients (59.5%) following R-PRRT, and in 5 patients (38.5%) and 7 patients (53.8%) following RR-PRRT, respectively. Median PFS was 14.6 months (95% CI 12.4–16.9) following R-PRRT and 14.2 months (95% CI 9.8–18.5) following RR-PRRT. Combined overall survival (OS) after I-PRRT plus R-PRRT and RR-PRRT was 80.8 months (95% CI 66.0–95.6). Grade III/IV bone marrow toxicity occurred in 6.6% and 7.7% of patients after R-PRRT and RR-PRRT, respectively. Salvage therapy resulted in a significantly longer OS in patients with bronchial NET, GEP-NET and midgut NET than in a nonrandomized control group. The total incidence of acute myeloid leukaemia (AML) and myelodysplastic syndrome (MDS) was 2.2%. No PRRT-related grade III/IV nephrotoxicity was observed.

**Conclusion:**

A cumulative dose of up to 60.5 GBq salvage PRRT with [^177^Lu-DOTA,Tyr^3^]octreotate is safe and effective in patients with progressive disease (relapse-PD) following I-PRRT with [^177^Lu-DOTA,Tyr^3^]octreotate. Safety appears similar to that of I-PRRT as no higher incidence of AML or MDS was observed. No grade III/IV renal toxicity occurred after retreatment.

**Electronic supplementary material:**

The online version of this article (10.1007/s00259-018-4158-1) contains supplementary material, which is available to authorized users.

## Introduction

Well-differentiated neuroendocrine tumours (NETs) are known to display a relatively indolent behaviour. Their current incidence is about 7 per 100,000 persons. There has been a gradual increase in their incidence over recent years [1]. Somatostatin analogues and the mammalian target of rapamycin (mTOR) inhibitor, everolimus, prolong progression-free survival (PFS) in patients with inoperable, advanced (metastatic) gastroenteropancreatic NET (GEP-NET), and sunitinib is approved for the treatment of advanced (metastatic) pancreatic NET [2–5]. However, recently the NETTER-1 study has demonstrated a superior outcome in terms of longer PFS (28.4 vs. 8.5 months) and overall survival (OS, ‘not reached’ after 42.0 months vs. 27.4 months) after peptide receptor radionuclide therapy (PRRT) with [^177^Lu-DOTA,Tyr^3^]octreotate plus 30 mg octreotide LAR every 4 weeks compared with high-dose octreotide LAR (60 mg every 4 weeks) in patients with advanced midgut NET, all previously treated with regular doses of octreotide LAR (20–30 mg every 4 weeks) [6, 7]. The US Food and Drug Administration (FDA) has therefore approved Lutathera® ([^177^Lu-DOTA,Tyr^3^]octreotate) for the treatment of somatostatin receptor-positive GEP-NET, including foregut, midgut and hindgut NETs, in adults. The European Medicines Agency (EMA) and the European Commission (EC) have also approved Lutathera® for the treatment of unresectable or metastatic, progressive, well-differentiated (grade I/II), somatostatin receptor-positive GEP-NET in adults.

For PRRT, high expression of somatostatin receptor subtypes on the surface of NET cells is required [8]. With respect to treatment outcome, initial PRRT (I-PRRT) with [^177^Lu-DOTA,Tyr^3^]octreotate has demonstrated objective radiological responses (ORR) in 29–39% and stabilization in 27–43% of patients with bronchial NET or GEP-NET [8–10]. The overall tolerability of PRRT is good; short-term and long-term side effects have been shown in the bone marrow and kidneys, but are mostly mild and reversible. There is a risk of approximately 2% for developing myeloproliferative disorders, including myelodysplastic syndrome (MDS) and acute myeloid leukaemia (AML) [8, 9, 11]. Eventually, most patients will demonstrate progressive disease (PD) after I-PRRT with limited treatment options left, particularly those with midgut NET. Interestingly, previous studies have indicated that salvage PRRT is feasible. Current data on salvage PRRT are mostly derived from studies with small patient cohorts and limited follow-up times. In some studies, different radioligands or their combinations have been used for I-PRRT and retreatment with PRRT (R-PRRT) [12–17].

Here we present the results in the largest cohort of patients from a single institution who underwent I-PRRT, R-PRRT and re-retreatment with PRRT (RR-PRRT) with only [^177^Lu-DOTA,Tyr^3^]octreotate and with the longest follow-up. Control groups consisting of patients not undergoing salvage therapy, but in principle qualifying for it, were established for comparison of median PFS after I-PRRT and OS in patients with bronchial NET or GEP-NET, and in the largest subgroups with midgut or pancreatic NET.

## Materials and methods

### Retreatment group selection

Dutch patients with advanced bronchial NET or GEP-NET who underwent R-PRRT or RR-PRRT between October 2003 and October 2015 were selected. Follow-up data were analysed until 1 January 2017. All included patients experienced PD (relapse-PD) after a period of disease control following I-PRRT with [^177^Lu-DOTA,Tyr^3^]octreotate. Disease control was defined as an objective response or stable disease (SD) according to the Response Evaluation Criteria in Solid Tumours 1.1 (RECIST 1.1). PD was based on CT or MRI or progression on [^111^In-DTPA^0^]octreotide scintigraphy (OctreoScan®). However, in a few patients salvage PRRT was started based on clinical deterioration without the requirement of qualifying for objective radiological progression. Relapse-PD is defined as the moment of PD before the start of R-PRRT and/or RR-PRRT, irrespective of known or objective PD before the start of I-PRRT. The same inclusion criteria as previously described for I-PRRT were applied for R-PRRT and RR-PRRT [8]. In brief, a Krenning tumour uptake score of at least grade II on the OctreoScan® and within the qualifying renal and haematological limits were required. In addition, only those patients with a PFS of ≥18.0 months from the first administration of I-PRRT were considered eligible for R-PRRT, and those with a PFS ≥14.0 months after R-PRRT for RR-PRRT.

### Nonrandomized control group selection

**Fig. 1:**
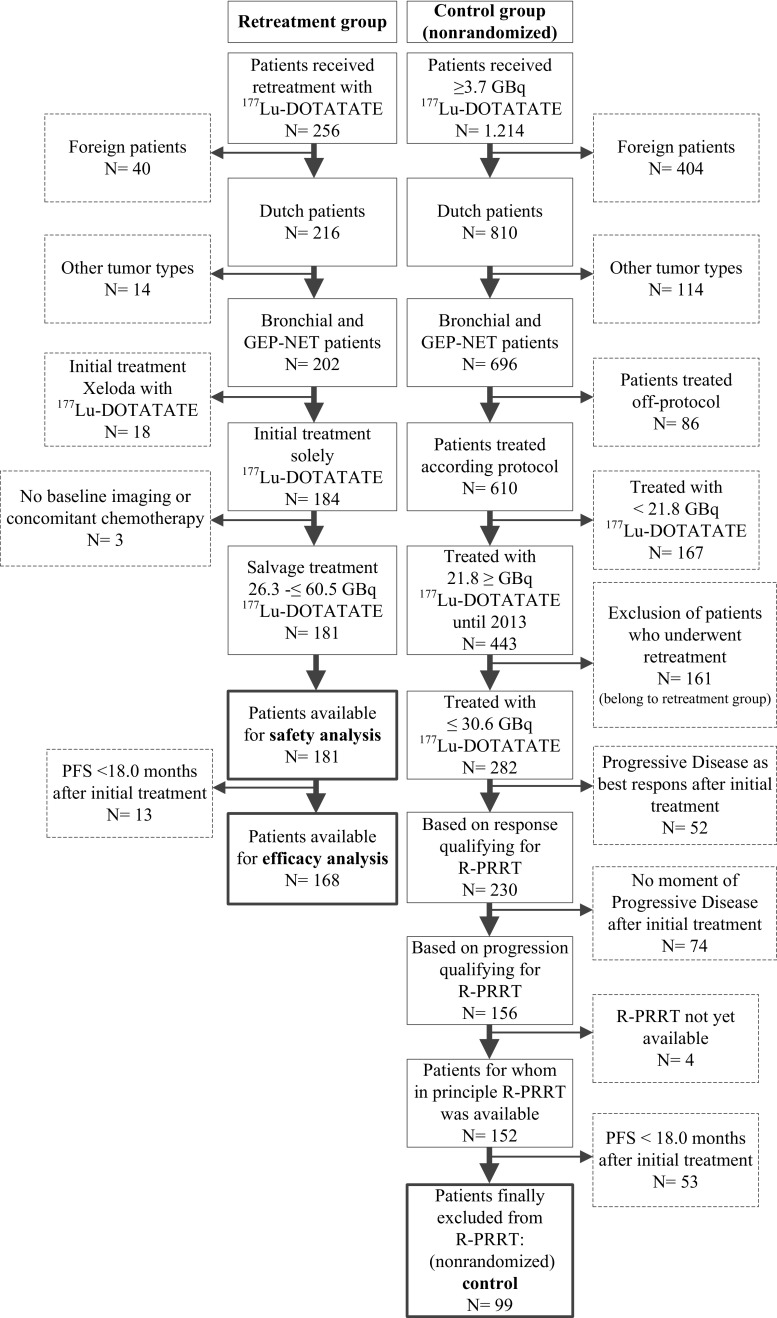
Flow chart showing selection of the retreatment and (nonrandomized) control groups. Boxes with *dashed lines* indicate reasons and numbers of patients excluded from analysis. Boxes with *bold lines* indicate total numbers of patients available for safety/efficacy analysis and comparison. See text for reasons of final exclusion

For estimation of a potential increase in OS, three nonrandomized but matched control groups were obtained, consisting of patients with bronchial and GEPNET, midgut NET or pancreatic NET. Group 1: bronchial and GEP-NET, Group 2: midgut NET, Group 3: pancreatic NET. These patients were treated and selected in our hospital and received 21.8–30.6 GBq [^177^Lu-DOTA,Tyr^3^]octreotate I-PRRT during the same study period and in principle could qualify for R-PRRT on the basis of the criteria described above. The reasons for control patients (99 patients) finally not receiving salvage PRRT were: insufficient uptake on OctreoScan® in progressive or newly developed lesions (2 patients); failure to meet renal and/or haematological inclusion parameters (25 patients); worsening of clinical condition (11 patients); receiving other local or systemic treatments (14 patients), lost to follow-up at the time of relapse-PD (9 patients); death (7 patients); SD after I-PRRT not considered by the treating investigator as favourable despite PFS ≥18.0 months (8 patients); wait-and-see strategy followed upon relapse-PD (2 patients); and development of a secondary primary tumour which required medical treatment (3 patients). In the remaining 18 control patients, it was unclear why R-PRRT was not considered. The flow chart presented in Fig. 1 shows the selection of the retreatment and nonrandomized control group, Fig. 1; the control group in this flow-chart is not divided in several groups, including the reasons for exclusion from analysis. Patients treated with 26.3– ≤60.5 GBq during R-PRRT or RR-PRRT were included in the safety analysis. Subgroups of patients with midgut NET or pancreatic NET were also separately analysed for efficacy.

### Methods

[DOTA,Tyr^3^]octreotate was obtained from BioSynthema. [^177^Lu-DOTA,Tyr^3^]octreotate was locally prepared as previously described [18]. To prevent nausea patients received a bolus of 3 mg granisetron intravenously. For kidney protection, an amino acid solution (2.5% arginine and 2.5% lysine in 1 L 0.225% NaCl) was infused over 4 h starting 30 min before administration of the radiopharmaceutical. The radiopharmaceutical itself was coadministered over 30 min via a separate infusion system. The administered dose per cycle was 7.4 GBq (or 3.7 GBq in patients with previous dose-modifying toxicity) with an intended cumulative dose of 29.6 GBq. No personalized dosimetry was applied for the salvage therapy. For R-PRRT and RR-PRRT the intended cumulative doses were 44.4 GBq and 59.2 GBq, respectively. The interval between treatments was 6 to 10 weeks.

Routine blood analysis for haematology, and liver and kidney function was performed 4–6 weeks after each cycle. During follow-up, CT or MRI was done at 6 weeks, 3 months and 6 months after the last treatment. Disease status was then evaluated biannually. Case report forms were completed at every hospitalization for treatment and follow-up visit. Safety was defined using the Common Terminology Criteria for Adverse Events (CTCAE), version 3.0.

This prospectively designed study with a retrospective analysis was approved by the medical ethics committee of our hospital and all patients gave written informed consent to their participation.

### Statistics

The correlations both between tumour response according to RECIST 1.1 after I-PRRT and response after R-PRRT and between PFS after I-PRRT and PFS after R-PRRT were tested using Spearman’s correlation coefficient. Medians and 95% confidence intervals (CI) for PFS and OS were estimated by the Kaplan-Meier method. Survival curves were compared using the log-rank test. Median follow-up time and median OS were calculated from the date of first administration of I-PRRT, and PFS from the date of first administration of I-PRRT, R-PRRT and RR-PRRT. Possible associations between changes in uptake on the OctreoScan® (Krenning score on baseline I-PRRT vs. the score on baseline R-PRRT OctreoScan®) and tumour response after R-PRRT were analysed using the chi-squared test. Groups were compared using Fisher’s exact test. The disease control rate (DCR) was defined as the proportion of patients who achieved a complete response (CR), partial response (PR) or SD. *P* values less than 0.05 were considered to indicate significant differences. Ranges are minimum and maximum values.

## Results

Baseline characteristics of patients who received I-PRRT, R-PRRT and RR-PRRT are shown in Table 1. Comparison of baseline parameters before I-PRRT and R-PRRT showed higher involvement of liver and/or bone metastases at the start of R-PRRT, reflecting progression of the disease over time. In total, seven patients had undergone debulking surgery after I-PRRT without disease progression at the time. During I-PRRT the retreatment and control patients received median cumulative doses of 29.9 GBq (range 18.6–30.7 GBq) and 29.8 GBq (range 21.8–30.6 GBq), respectively. Of 168 patients who underwent R-PRRT, 153 received the intended additional dose, and all patients who underwent RR-PRRT received the intended dose. Patients received a median cumulative administered dose of 14.9 GBq (3.7–16.2 GBq) and 15.0 GBq (14.7–15.3 GBq), resulting in total median cumulative administered doses of 44.7 GBq (26.3–46.4 GBq) after R-PRRT and 59.7 GBq (55.2–60.5 GBq) after RR-PRRT. The main reasons for not completing the intended number of cycles were persistent myelotoxicity and clinical deterioration (Table 2). From the start of I-PRRT, the overall median follow-up time was 88.6 months (95% CI 79.0–98.2 months), including median follow-up times of 30.4 months (95% CI 22.5–38.4 months) and 34.5 months (95% CI 16.5–52.5 months) from the start of R-PRRT and RR-PRRT, respectively.

**Table 1:** Baseline characteristics of patients with bronchial NET or GEP-NET prior to I-PRRT, R-PRRT and RR-PRRT with [^177^Lu-DOTA,Tyr^3^]octreotate

Characteristic	Before I-PRRT (*n* = 168)	Before R-PRRT (*n* = 168)	Before RR-PRRT (*n* = 13)	*p* value^a^
Male, *n* (%)	95 (56.5)	95 (56.5)	9 (69.2)	
Age (years), median (range)	59 (32–78)	63 (35–80)	64 (46–78)	
Tumour type, *n* (%)				
Bronchial NET	13 (7.7)	13 (7.7)	2 (15.4)	
Pancreatic NET	53 (31.5)	53 (31.5)	4 (30.8)	
Midgut NET	54 (32.1)	54 (32.1)	2 (15.4)	
Baseline progression, *n* (%)				
According RECIST 1.1				
Yes	98 (58.3)	152 (90.5)	11 (84.6)	<0.01
No	22 (13.1)	2 (1.2)	1 (7.7)	
Unknown	48 (28.6)	–	–	
On OctreoScan®				
Yes	–	13 (7.7)	1 (7.7)	
Clinical symptoms				
Yes	–	1 (0.6)	0 (0.0)	
Prior treatment, *n* (%)				
Surgery				
Yes	71 (42.3)	7 (4.2)^b^	0 (0.0)	
No	97 (57.7)	161 (95.8)	13 (100)	
Chemotherapy				
Yes	9 (5.4)	–	–	
No	159 (94.6)	–	–	
Radiotherapy				
Yes	12 (7.1)	–	–	
No	155 (92.3)	–	–	
Unknown	1 (0.6)	–	–	
Somatostatin analogues				
Yes	98 (58.3)	–	–	
No	70 (41.7)	–	–	
Extent of disease, *n* (%)^c^				
Limited	23 (13.7)	23 (13.9)	1 (7.7)	1.00
Moderate	122 (72.6)	83 (50.0)	7 (53.8)	<0.01
Extensive	23 (13.7)	60 (36.1)	5 (38.5)	<0.01
Unknown	–	2 (−)	0 (–)	
Uptake on OctreoScan®, *n* (%)				
Score 2	9 (5.4)	14 (8.4)	0 (0.0)	0.39
Score 3	93 (55.4)	91 (54.8)	5 (38.5)	0.66
Score 4	66 (39.3)	61 (36.7)	8 (61.5)	0.65
Unknown	–	2 (−)	0 (–)	
Liver lesions, *n* (%)				
Yes	153 (91.1)	163 (97.0)	13 (100)	0.04
No	15 (8.9)	5 (3.0)	0 (0.0)	
Bone lesions, *n* (%)				
Yes	47 (28.0)	74 (44.0)	7 (53.8)	<0.01
No	121 (72.0)	94 (56.0)	6 (46.2)	
Chromogranin A				
>2 × ULN, *n* (%)	113 (70.2)	117 (70.5)	9 (69.2)	0.48
Serum level (μg/L), median (Q_1_–Q_3_)	442 (148–2,027)	462 (117–2,300)	363 (128–1,007)	
Unknown, *n* (%)	7 (−)	2 (−)	0 (0)	
Alkaline phosphatase				
>2 × ULN, *n* (%)	25 (15.5)	35 (21.0)	2 (15.4)	0.20
Serum level (U/L), median (Q_1_–Q_3_)	108 (78–159)	110 (78–172)	95 (84–151)	
Unknown, *n* (%)	4 (−)	0 (−)	0 (−)	
WHO tumour grade, *n* (%)^d^				
I	22 (22.7)	–	–	
II	66 (68.0)	–	–	
III	9 (9.3)	–	–	
Unknown	71 (−)	–	–	

Patients of the I-PRRT and R-PRRT groups are the same

*ULN* upper limit of normal, *Q*_*1*_*–Q*_*3*_ interquartile range

^a^I-PRRT vs. R-PRRT

^b^Surgery after I-PRRT and before R-PRRT

^c^Represents regional distribution of metastatic spread on OctreoScan® as described previously [22]

^d^Most patients with an unknown Ki-67 proliferation index were treated before 2007. After 2007 the Ki-67 proliferation index was routinely checked by MIB-1 staining. Before the start of R-PRRT/RR-PRRT the Ki-67 index was not routinely checked

**Table 2:** Reasons for not achieving the intended cumulative dose of 14.8 GBq during R-PRRT

Reason	Number of patients (*n* = 15)
Persistent myelosuppression	4^a^
Clinical deterioration	4
Objective progressive disease	1
Death after first cycle	3
Development of breast cancer, start of another treatment	1
Stomach ulcer caused by NET, coiling necessary due to severe melaena and haematemesis	1
Diffuse bone metastases (started with reduced dose)	1

These patients were included in the efficacy analysis

^a^Thrombocytopenia grade II in two patients, grade III in two patients

**Fig. 2:**
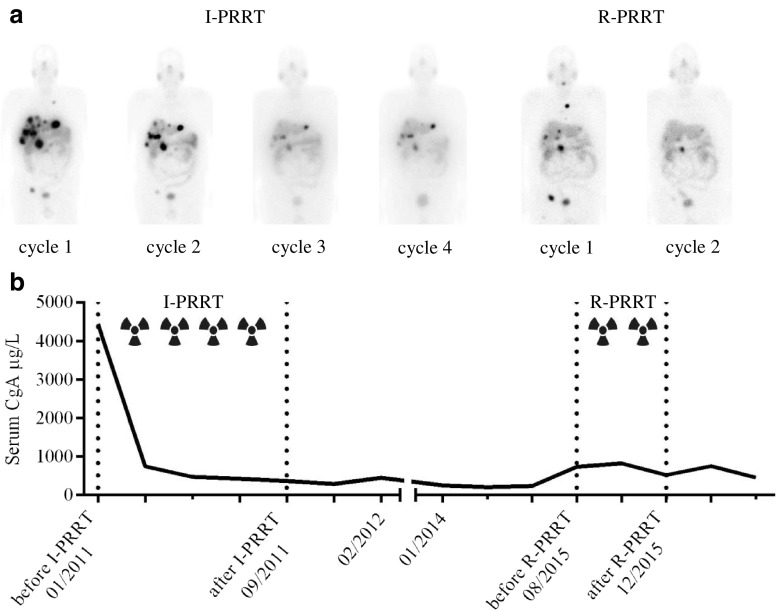
Planar scintigraphy in a patient with metastasized well-differentiated pancreatic NET. **a** Images obtained 24 h after administration of 7.4 GBq of [^177^Lu-DOTA^0^,Tyr^3^]octreotate during the initial four cycles (I-PRRT) and two additional cycles (R-PRRT) after PD in the first half of 2015 show high uptake by somatostatin receptor-positive tumours. **b** Course of the tumour marker chromogranin A (CgA) during and after treatment with PRRT

The efficacy of R-PRRT was evaluated in 168 patients and of RR-PRRT in 13 patients with bronchial NET or GEP-NET. Radiological tumour responses at 3 months and the best responses after the last treatment cycle are shown in Table 3. R-PRRT resulted in an ORR of 15.5% and SD of 59.5%. A total of 33 patients (19.6%) had objective disease progression as best response after R-PRRT, and of these 54.5% had a PR and 45.5% SD as best response after I-PRRT. Five patients (3.0%) died before the intended cumulative dose of 14.8 GBq was given. Three patients (1.8%) showed clinical deterioration and were considered as having PD during therapy as well. Of 13 RR-PRRT patients, 5 (38.5%) achieved an ORR and 7 (53.8%) achieved SD, and 1 (7.7%) had PD. In the 167 patients in the retreatment group, radiological tumour responses to R-PRRT and to I-PRRT were significantly correlated (*r*_S_ = 0.259, *p* < 0.01; data not shown). Figure 2 shows a patient with metastasized well-differentiated pancreatic NET achieving a PR after both I-PRRT and R-PRRT.

**Table 3:** Radiological tumour response evaluation in patients with bronchial NET or GEP-NET

	Number of patients	Best response				NE	Clinical PD	Response at 3 months follow-up			NE	Clinical PD	Died before start of follow-up
		CR	PR	SD	PD^a^			PR	SD	PD			
After I-PRRT													
Control group	99	0 (0.0)	36 (36.4)	58 (58.6)	–	5 (5.1)	–	–	–	–	–	–	–
Salvage group	168	1 (0.6)	93 (55.4)	73 (43.5)	–	1 (0.6)	–	–	–	–	–	–	–
After salvage PRRT													
R-PRRT	168	–	26 (15.5)	100 (59.5)	33 (19.6)	1 (0.6)	3 (1.8)	14 (8.3)	111 (66.1)	34 (20.2)	1 (0.6)	3 (1.8)	5 (3.0)
RR-PRRT	13	–	5 (38.5)	7 (53.8)	1 (7.7)	–	–	2 (15.4)	9 (69.2)	2 (15.4)	–	–	–

The data are presented as number (%) of patients

Patients with PD as best response after I-PRRT were considered as having treatment failure and were not eligible for subsequent R-PRRT

*CR* complete response, *PR* partial response, *SD* stable disease, *PD* progressive disease, *NE* not evaluable

^a^Salvage patients were those with PD after I-PRRT and an initial tumour response of at least SD lasting for at least 18 months from the first administration of the I-PRRT

Irrespective of the tumour response after I-PRRT, after R-PRRT the median PFS was 14.6 months (95% CI 12.4–16.9 months) and after RR-PRRT was 14.2 months (95% CI 9.8–18.5 months). However, patients who had SD and PR after R-PRRT (75%) had a median PFS of 19.4 months (95% CI 17.2–21.5 months). Seven out of nine patients (78%) with WHO tumour grade III (Ki-67 index 20–30%) responded to R-PRRT (six patients with SD and one with PR), and had a median PFS of 13.4 months (95% CI 10.9–15.8 months). The two patients with PD as best tumour response were omitted from this PFS analysis. For the entire group of 168 patients, PFS after I-PRRT and after R-PRRT were significantly associated (*r*_S_ = 0.325, *p* < 0.01; Online Resource 1).

Between the baseline I-PRRT and baseline R-PRRT the OctreoScan® tumour uptake score increased in 27 patients (16.3%) and decreased in 33 patients (19.9%) (an extended overview of these findings is given in Online Resource 2). Only 5 of 33 patients (one patient not evaluable) with PD as response after R-PRRT had a lower tumour uptake score at the start of R-PRRT compared with I-PRRT. No relationship was found between the changes in tumour uptake on OctreoScan® and tumour response after R-PRRT: *χ*^2^ = 3.47 (degrees of freedom 6, *n* = 165), *p* = 0.75.

OS was compared between the matched, nonrandomized control group of patients with bronchial NET or GEP-NET after receiving I-PRRT followed by unknown therapies and the retreatment groups after receiving I-PRRT, R-PRRT, RR-PRRT and unknown consecutive therapies. The baseline characteristics of the control and retreatment groups are shown in Table 4. Both groups were balanced with respect to baseline progression, prior treatments, extent of disease and number of patients with increased serum chromogranin A levels. More patients in the retreatment group had liver metastases (81.8% of patients in the control group vs. 91.1% in the retreatment group) and bone lesions (12.1% vs. 28.0%, respectively). A different distribution of tumour uptake was seen on OctreoScan® (uptake score 3 in 72.7% of patients in the control group and in 55.4% in the retreatment group) and tumour grade (WHO grade II in 42.9% of patients in the control group and in 68.0% in the retreatment group). For grading we assumed a similar Ki-67 distribution in our patient population before and after 2007, the year measurement of this parameter was introduced in our institution.

**Table 4:** Baseline characteristics of patients with bronchial NET and GEP-NET in the control group and the retreatment group before any PRRT

Characteristic	Control group (*n* = 99)	Retreatment group (*n* = 168)	*p* value
Male, *n* (%)	45 (45.5)	95 (56.5)	
Age (years), median (range)	64 (37–83)	59 (32–78)	
Baseline progression, *n* (%)^a^			
Yes	46 (46.5)	98 (58.3)	0.08
No	21 (21.2)	22 (13.1)	
Unknown	32 (32.3)	48 (28.6)	
Prior treatment, *n* (%)			
Surgery			
Yes	43 (43.4)	71 (42.3)	0.90
No	56 (56.6)	97 (57.7)	
Chemotherapy			
Yes	6 (6.1)	9 (5.4)	0.79
No	93 (93.9)	159 (94.6)	
Radiotherapy			
Yes	3 (3.0)	12 (7.1)	0.18
No	96 (97.0)	155 (92.3)	
Unknown	–	1 (0.6)	
Somatostatin analogues			
Yes	54 (54.5)	98 (58.3)	0.61
No	45 (45.5)	70 (41.7)	
Extent of disease, *n* (%)^b^			
Limited	8 (8.1)	23 (13.7)	0.23
Moderate	76 (76.8)	122 (72.6)	0.47
Extensive	15 (15.2)	23 (13.7)	0.06
Uptake on OctreoScan®, *n* (%)			
Score 2	0 (0.0)	9 (5.4)	0.03
Score 3	72 (72.7)	93 (55.4)	0.01
Score 4	27 (27.3)	66 (39.3)	0.06
Liver lesions, *n* (%)			
Yes	81 (81.8)	153 (91.1)	0.03
No	18 (18.2)	15 (8.9)	
Bone lesions, *n* (%)			
Yes	12 (12.1)	47 (28.0)	<0.01
No	87 (87.9)	121 (72.0)	
Chromogranin A			
>2 × ULN, *n* (%)	68 (72.3)	113 (70.2)	0.89
Serum level (μg/L), median (Q_1_–Q_3_)	405 (165–2,025)	442 (148–2,027)	
Unknown, *n* (%)	5 (−)	7 (−)	
Alkaline phosphatase			
>2 × ULN, *n* (%)	14 (14.1)	25 (15.2)	1.00
Serum level (U/L), median (Q_1_–Q_3_)	110 (80–158)	108 (78–159)	
Unknown, *n* (%)	0 (−)	4 (−)	
WHO tumour grade, *n* (%)^c^			
I	15 (53.5)	22 (22.7)	0.71
II	12 (42.9)	66 (68.0)	<0.01
III	1 (3.6)	9 (9.3)	0.10
Unknown	71 (−)	71 (−)	
Tumour response, *n* (%)^d^			
CR	0 (0.0)	1 (0.6)	1.00
PR	36 (38.3)	93 (55.7)	< 0.01
SD	58 (61.7)	73 (43.7)	0.02
Unknown	5 (−)	1 (−)	

*ULN* upper limit of normal, *Q*_*1*_*–Q*_*3*_ interquartile range, *CR* complete response, *PR* partial response, *SD* stable disease

^a^Documented progression according RECIST 1.1

^b^Represents regional distribution of metastatic spread on OctreoScan® as described previously [22]

^c^Since 2007 the Ki-67 proliferation index was routinely checked by MIB-1 staining. Most patients with an unknown Ki-67 index were treated before 2007

^d^Tumour response to I-PRRT evaluated according RECIST 1.1

The efficacy of salvage therapy was compared between 168 patients receiving R-PRRT and 99 control patients. OS and PFS in the various treatment groups in relation to tumour classification are shown in Table 5. In terms of PFS, the control group and retreatment group (bronchial NET or GEP-NET) were well matched. However, the proportions of patients with midgut NET (*n* = 63) and pancreatic NET (*n* = 20) were unevenly distributed in the control group and therefore subgroup analyses of patients with midgut NET or pancreatic NET were also performed. Baseline characteristics of the patients with midgut NET or pancreatic NET are shown in Online Resource 3 and Online Resource 4.

**Table 5:** PFS, OS and median follow-up time in patients receiving I-PRRT, R-PRRT and RR-PRRT with [^177^Lu-DOTA,Tyr^3^]octreotate and in the control group

	I-PRRT/R-PRRT			RR-PRRT		Control group		*p* value
	Number (%) of patients	Median (95% CI)		Number (%) of patients	Median (95% CI)	Number (%) of patients	Median (95% CI)	
		I-PRRT	R-PRRT					
Follow-up (months)^a^		88.6 (79.0–98.2)	30.4 (22.5–38.4)		34.5 (16.5–52.5)		120.2 (90.9–149.5)	
PFS (months)^a^								
All tumours	168 (100)	35.4 (33.0–37.8)	14.6 (12.4–16.9)	13 (100)	14.2 (9.8–18.5)	99 (100)	35.5 (30.3–40.7)	0.86^b^
Foregut NET	5 (3.0)	51.6 (44.0–59.3)	14.6 (N/A)	1 (7.7)	13.8 (N/A)			
Midgut NET	54 (32.1)	40.8 (31.1–50.5)	14.7 (8.9–20.4)	2 (15.4)	4.7 (N/A)	63 (63.3)	35.5 (31.4–39.7)	0.41^b^
Hindgut NET	12 (7.1)	29.4 (11.2–47.6)	20.0 (0.6–39.4)	1 (7.7)	13.5 (N/A)			
Pancreatic NET	53 (31.5)	32.7 (27.2–38.1)	14.4 (11.5–17.2)	4 (30.8)	19.3 (10.4–28.1)	20 (20.2)	39.1 (31.8–46.5)	0.59^b^
Bronchial NET	13 (7.7)	25.4 (18.9–31.9)	8.0 (4.5–11.6)	2 (15.4)	12.0 (N/A)			
Unknown NET	31 (18.8)	36.2 (28.2–44.3)	13.6 (9.6–17.6)	3 (23.1)	17.6 (1.4–33.8)			
OS (months)^a^								
All tumours	168 (100)	80.8^c^ (66.0–95.6)	26.2 (17.9–34.5)	13 (100)	39.5 (14.4–64.6)	99 (100)	51.4^d^ (46.7–56.2)	<0.01^e^
Foregut NET	5 (3.0)	81.3 (56.9–105.7)	33.9 (N/A)	1 (7.7)	18.3 (N/A)			
Midgut NET	54 (32.1)	77.3 (55.0–99.5)	23.1 (18.4–27.9)	2 (15.4)	29.6 (N/A)	63 (63.6)	51.0 (44.6–57.4)	<0.01^e^
Hindgut NET	12 (7.1)	97.8 (31.8–163.4)	56.9 (8.1–105.6)	1 (7.7)	17.1 (N/A)			
Pancreatic NET	53 (31.5)	93.9 (39.4–148.3)	36.8 (20.8–52.8)	4 (30.8)	44.7 (36.4–53.1)	20 (20.2)	61.5 (49.9–73.2)	0.57^e^
Bronchial NET	13 (7.7)	74.7 (22.6–126.8)	26.2 (0.0–52.5)	2 (15.4)	17.8 (N/A)			
Unknown NET	31 (18.8)	77.7 (43.3–112.2)	22.2 (12.7–31.7)	3 (23.1)	20.8 (13.5–28.1)			

^a^Median follow-up time, PFS and OS were calculated from the date of the first administration of I-PRRT, R-PRRT and RR-PRRT

^b^Control group vs. I-PRRT (excluding additional PFS due to R-PRRT or RR-PRRT)

^c^Total OS for I-PRRT, R-PRRT, RR-PRRT and unknown consecutive therapies combined

^d^Total OS for I-PRRT and unknown consecutive therapies

^e^Control group vs. I-PRRT, R-PRRT and RR-PRRT combined

**Fig. 3:**
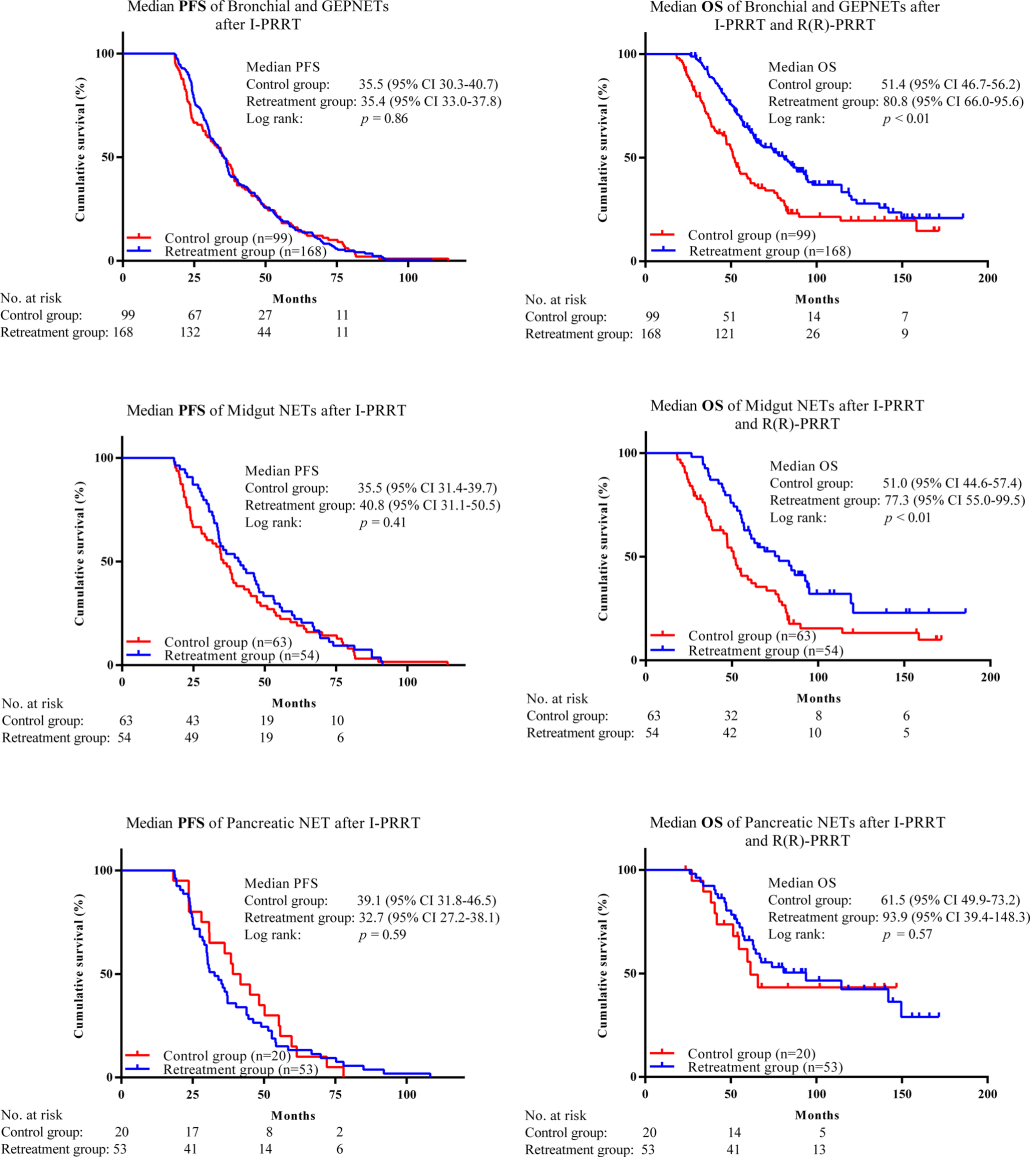
PFS and OS in patients with bronchial NET and GEP-NET, midgut NET and pancreatic NET and in patients in the respective control groups. The PFS of the R-PRRT group, for comparison with the PFS in the control group, was obtained after I-PRRT, and thus does not include the additional PFS due to R-PRRT or RR-PRRT. The two groups had comparable PFS. The OS of the control group was obtained after I-PRRT plus unknown other treatment(s) and is compared with the cumulative OS in patients receiving I-PRRT, R-PRRT, RR-PRRT and unknown other treatment(s) obtained after the last salvage PRRT

Patients with bronchial NET or GEP-NET treated with salvage PRRT had a significantly longer OS than control patients (*p* < 0.01). The combined median OS in patients receiving I-PRRT, R-PRRT and RR-PRRT was 80.8 months (95% CI 66.0–95.6), and in the control patients was 51.4 months (95% CI 46.7–56.1 months). In patients with midgut NET, OS was 77.3 months (95% CI 55.0–99.5 months) in the retreatment group and 51.0 months (95% CI 44.6–57.4 months) in the control patients. In patients with pancreatic NET there was a trend towards improved OS following salvage PRRT: OS was 93.9 months (95% CI 39.4–148.3 months) in the retreatment group and 61.5 months (95% CI 49.9–73.2 months) in the control patients (*p* not significant; Fig. 3).

Safety was evaluated in 181 R-PRRT patients and 13 RR-PRRT patients. Grade III/IV subacute haematological toxicity occurred in 12 R-PRRT patients (6.6%) and in 1 RR-PRRT patient (7.7%; Table 6). Myeloproliferative toxicity in the R-PRRT and control patients was not significantly different (2.2% and 3.5%, respectively). AML was diagnosed in two patients and MDS in two patients after R-PRRT (Table 7). No further patients were diagnosed with AML/MDS after RR-PRRT. No PRRT-related grade III/IV nephrotoxicity was observed.

**Table 6:** PRRT-related subacute haematological toxicities in the control and retreatment groups

Toxicity^a^	Control group (*n* = 230)		I-PRRT (*n* = 181)		R-PRRT (*n* = 181)		RR-PRRT (*n* = 13)		*p* value	
	Grade III	Grade IV	Grade III	Grade IV	Grade III	Grade IV	Grade III	Grade IV	Control vs. I-PRRT	I-PRRT vs. R-PRRT
Any	30 (13.0%)	6^b^ (2.6%)	19 (10.5%)	1 (0.6%)	12 (6.6%)	1 (0.6%)	1 (7.7%)	0 (0.0%)	0.45 (grade III toxicities); 0.14 (grade IV toxicities)	0.26
Haemoglobin	13	0	2	1	1	1	1	0		1.00
Leucocytes	16	1	14	0	6	0	0	0		
Platelets	14	5	6	0	5	0	0	0		
Creatinine	1	0	0	0	0	0	0	0		

^a^Graded according CTCAE 3.0 during complete follow-up

^b^Five patients had concurrent grade III toxicities

**Table 7:** PRRT-related myeloproliferative toxicities in the control and retreatment groups

	Control group (*n* = 230)	Retreatment group (*n* = 181)	*p* value
Any	8 (3.5%)	4 (2.2%)	0.56
AML	2	2	
MDS	6	2	
Time to diagnosis (months), median (95% CI)			
From start of I-PRRT	28.7 (26.9–30.5)	33.8 (16.8–50.9)	0.14
From start of R-PRRT	–	11.5 (1.8–21.2)	

*AML* acute myeloid leukaemia, *MDS* myelodysplastic syndrome

## Discussion

We report here the safety and efficacy of salvage PRRT in the largest series of patients receiving [^177^Lu-DOTA,Tyr^3^]octreotate to a cumulative administered dose of up to 60.5 GBq, and with the longest follow-up period [14, 16]. The long follow-up period enabled the estimation of both PFS and OS after salvage PRRT. Current literature on salvage therapy includes studies with the same, other or combinations of radiolabelled somatostatin analogues: [^177^Lu-DOTA,Tyr^3^]octreotide after [^90^Y- DOTA,Tyr^3^]octreotide in 27 patients [12]; only [^177^Lu-DOTA,Tyr^3^]octreotate in 33 patients [16], in 33 patients [14] and in 14 patients [17]; a mixture of [^90^Y-DOTA,Tyr^3^]octreotate, [^177^Lu-DOTA,Tyr^3^]octreotate, and [^177^Lu/^90^Y-DOTA,Tyr^3^]octreotate in 16 patients [13]; and [^177^Lu-DOTA,Tyr^3^]octreotate after [^90^Y- DOTA,Tyr^3^]octreotide in 26 patients [15]. Safety profiles of PRRT with ^177^Lu-labelled and ^90^Y-labelled somatostatin analogues differ, and in particular renal toxicity is more often reported after the use of ^90^Y-labelled compounds [11, 19]. In this study, we focused only on the results of [^177^Lu-DOTA,Tyr^3^]octreotate-based I-PRRT and R-PRRT.

Sabet et al. retrospectively analysed 33 patients (including 14 with advanced pancreatic NET and 6 with advanced midgut NET) with WHO grade I/II metastatic GEP-NET retreated with [^177^Lu-DOTA,Tyr^3^]octreotate after initial [^177^Lu-DOTA,Tyr^3^]octreotate therapy [14]. The cumulative mean dose of [^177^Lu-DOTA,Tyr^3^]octreotate after R-PRRT was 44.3 GBq (range 30–83.7 GBq). The median follow-up times were 37 and 23 months from the start of I-PRRT and R-PRRT, respectively. Analysis in relation to tumour type was not reported. Tumour responses according to Southwest Oncology Group criteria after salvage included 7 patients (21.2%) with ORR, 15 (45,4%) with SD, and 11 (33.3%) with PD, with a mean PFS of 13 months (data on OS were not reported). Reversible grade III/IV haematotoxicity was recorded in 7 patients (21.2%), without any MDS or grade III/IV renal toxicity [14]. Yordanova et al. from the same institution recently reported the results in 15 patients with advanced GEP-NET retreated with [^177^Lu-DOTA,Tyr^3^]octreotate. The highest administered dose of [^177^Lu-DOTA,Tyr^3^]octreotate (96.6 GBq) led to bone marrow and kidney absorbed radiation doses of 6.8 and 87 Gy, respectively, but without inducing any grade III/IV toxicity in these organs, after a median follow-up of 62 months (range 31–134 months) [17].

The 33 patients in our earlier salvage study reported in 2010 [16] were also included in this expanded study, which led to a doubling of the median follow-up time to 88.6 months. The original reported time-to-progression following the first cycle of retreatment with [^177^Lu-DOTA,Tyr^3^]octreotate was 17 months, which was based only on patients with ORR and SD, thereby excluding those with PD.

In the present study, additional cycles of [^177^Lu-DOTA,Tyr^3^]octreotate were well tolerated in the majority of patients. R-PRRT had to be stopped as a precaution in only four patients because of persistent myelosuppression. In two of these patients the maximum cumulative administered dose for I-PRRT was not reached. One of these four patients developed MDS, which was diagnosed 1.7 months after the first (and only) administration of R-PRRT. In our view, 1.7 months is too short as latency period after the start of retreatment for the R-PRRT to have been the cause of the MDS. Additionally, blood transfusion because of grade III haemoglobin toxicity was needed before R-PRRT. We therefore hypothesize that I-PRRT was already and mainly releated to the development of MDS. However, no factor is known to be able to predict which patient is prone to the development of such a serious bone marrow complication [20]. Three of the four patients affected by MDS and AML had no dose-modifying toxicities during I-PRRT or R-PRRT. None of these four patients had been pretreated with chemotherapy. There was no indication of increased renal or haematological toxicity after administration of cumulative doses of [^177^Lu-DOTA,Tyr^3^]octreotate higher than those administered during I-PRRT.

Of the patients in the retreatment group, 56.0% showed an ORR of and 43.5% SD after I-PRRT (those with PD after I-PRRT were not candidates for R-PRRT and were excluded). In this respect, an objective best response rate of 15.5% with an ORR and 59.5% with SD following R-PRRT seems on first sight to be a worse response than after I-PRRT. However, the administered dose of [^177^Lu-DOTA,Tyr^3^]octreotate in R-PRRT was only half of that used in I-PRRT, leading to a lower radiation dose to the tumours. Also, the more advanced disease state at the start of R-PRRT might have led to a lower tumour response rate following [^177^Lu-DOTA,Tyr^3^]octreotate treatment. More extensive disease is associated with shorter OS [9]. The overall changes in tumour uptake scores, as assessed on planar OctreoScan® scintigraphy before I-PRRT and RR-PRRT with [^177^Lu-DOTA,Tyr^3^]octreotate, were not statistically significant, and therefore changes in tumour uptake seem to be poorly associated with the responses seen after R-PRRT.

It is not surprising that a lower dose (e.g. retreatment with a cumulative administered dose of 14.8 GBq [^177^Lu-DOTA,Tyr^3^]octreotate) resulted in a trend towards fewer side effects than a cumulative administered dose of 29.6 GBq. Retreatment with 14.8 GBq in two cycles may therefore not be considered as the maximum tolerated administered activity for retreatment. The present promising efficacy results of salvage PRRT with only two cycles of [^177^Lu-DOTA,Tyr^3^]octreotate do not imply that, a priori, these two salvage cycles should be automatically increased to four cycles for retreatment, as used during I-PRRT. The DCR of 75.0% after two cycles of R-PRRT is already very encouraging and RR-PRRT with two cycles after relapse (PFS of 14.2 months) is likewise effective and safe.

PFS was 14.6 months in the entire group of patients with bronchial NET or GEP-NET after R-PRRT with [^177^Lu-DOTA,Tyr^3^]octreotate and was 19.4 months when PR and SD were considered as tumour responses. PFS was 14.7 months in patients with midgut NET and 14.4 months in patients with pancreatic NET after R-PRRT. The fact that PFS after I-PRRT and after R-PRRT were correlated supports the use of PFS as a patient selection criterion for retreatment. It should be emphasized that this correlation was found in our study using a minimum PFS of 18.0 months as the selection criterion for salvage therapy. Sabet et al. similarly found a more durable PFS after R-PRRT in patients with a longer PFS after I-PRRT [14].

Currently, targeted therapies such as everolimus and sunitinib are available as alternative options for I-PRRT, R-PRRT and RR-PRRT, and thus as second-line and third-line therapies. Everolimus is currently approved for the treatment of advanced, progressive, NETs of the pancreas, gastrointestinal tract and lung, whereas sunitinib is only approved for the treatment of advanced progressive pancreatic NETs. In the RADIANT-4 study patients with progressive, nonfunctional grade I/II gastrointestinal or lung NETs were treated with everolimus, and showed a PFS of 11.0 months (95% CI 9.2–13.3) [21]. In patients with advanced, progressive, low-grade or intermediate-grade pancreatic NETs PFS was comparable between those treated with everolimus and those treated with sunitinib: 11.0 months [5] and 11.4 months [3], respectively. Since the maximum tolerated administered dose of [^177^Lu-DOTA,Tyr^3^]octreotate has yet not been reached, it may be argued that these targeted therapies should be reserved for patients with a relapse-PD after salvage PRRT with [^177^Lu-DOTA,Tyr^3^]octreotate.

The NETTER-1 study introduced a form of salvage therapy in which the octreotide LAR dose was increased by a factor of two or three to 60 mg every 4 weeks in patients with midgut NET, all of whom had PD following previous octreotide LAR treatment [6]. The median PFS was 8.4 months in the control arm treated with salvage octreotide LAR therapy, substantially lower than in the patients with midgut NET in the present study who received R-PRRT. These findings may be of importance in deciding the sequence in the use of octreotide LAR, including salvage therapy, and subsequent PRRT with [^177^Lu-DOTA,Tyr^3^]octreotate including salvage therapy.

The OS after salvage PRRT was compared with the OS in the control group with bronchial NET or GEP-NET. These two groups were more or less matched with respect to many baseline characteristics. Despite the high frequency of poor prognostic parameters, e.g. the presence of liver and bone metastases in our salvage PRRT group, we still found a remarkable OS of more than 80 months after R-PRRT and RR-PRRT combined. Salvage therapy with [^177^Lu-DOTA,Tyr^3^]octreotate) resulted in an OS almost 30 months longer than in the control group. However, the numbers of patients with midgut NET (63 patients) and pancreatic NET (20 patients) in the control group were unevenly distributed compared with the respective retreatment groups. Therefore, subgroups of patients with midgut NET and pancreatic NET were formed for both the control and retreatment patient groups, and these were also separately analysed for tumour response, PFS and OS. The PFS in the patients with midgut NET and pancreatic NET in the retreatment subgroups matched the PFS following I-PRRT in the controls. Patients with midgut NET showed a significantly longer OS of more than 21 months after retreatment. However, patients with pancreatic NET showed a trend towards a longer OS with a survival benefit of approximately 32 months, although this was not significantly different. This might have been related to the availability of only a small number of patients for this control group and the availability of various alternative treatment options specifically for patients with pancreatic NET.

PRRT is mostly used in patients with well or moderately differentiated NET, but PRRT can be effective in selected patients with poorly differentiated neuroendocrine carcinomas (NECs). In our series, nine patients with grade III NEC (Ki-67 index 20–30%) underwent R-PRRT. Seven of these nine patients had a favourable tumour response to I-PRRT (four with PR and four with SD) and also to R-PRRT (one with PR and six with SD). It is possible that these patients were selected because of a relatively indolent (for NEC) disease course. Thus, R-PRRT seems to be a feasible option in a subgroup of patients with GEP-NEC, primarily in those with a Ki-67 index below 30%.

We present data from the Rotterdam cohort on the efficacy and safety of salvage treatment with [^177^Lu-DOTA,Tyr^3^]octreotate in patients with relapse-PD after benefiting from I-PRRT. This patient group was compared with a nonrandomized control group with bronchial NET or GEP-NET, including a similar subgroup analysis of patients with midgut NET and pancreatic NET. The feasibility of R-PRRT has previously been reported, but the strengths of this study lie in the selection of both retreatment patients and non-randomized control patients with bronchial NET or GEP-NET and their subgroups of patients with midgut NET and pancreatic NET, the very substantial follow-up time carried out in a single institution, the large patient cohort size, and the use of only [^177^Lu-DOTA,Tyr^3^]octreotate. In this study of salvage therapy with [^177^Lu-DOTA,Tyr^3^]octreotate up to an administered cumulative dose of 60.5 GBq, no personalized dosimetry was used, but instead treatment dosing relied on the usual follow-up parameters of bone marrow and renal function. The safe toxicity profile found with this procedure calls into question the added value of personalized dosimetry [22].

We underline the necessity for randomized controlled trials to compare the efficacy and safety of retreatment with [^177^Lu-DOTA,Tyr^3^]octreotate and approved targeted therapies (e.g. everolimus and sunitinib).

### Conclusion

Salvage PRRT with [^177^Lu-DOTA,Tyr^3^]octreotate to a cumulative administered dose of up to 60.5 GBq can be performed safely and effectively after progression following I-PRRT with [^177^Lu-DOTA,Tyr^3^]octreotate. The safety is similar to that after I-PRRT. In particular, the incidence of AML and MDS was not higher and renal toxicity grade III/IV was not observed. Salvage therapy resulted in a significantly longer OS in patients with bronchial NET, GEP-NET or midgut NET than in a nonrandomized control group. In patients with pancreatic NET, the OS was not significantly different, but there seemed to be a trend towards improvement in OS after salvage therapy.

## Electronic supplementary material

Online Resource 1 (DOCX 138 kb)

Online Resource 2 (DOCX 31 kb)

Online Resource 3 (DOCX 24 kb)

Online Resource 4 (DOCX 25 kb)
