# Supplementary material for: Salvage peptide receptor radionuclide therapy with [177Lu-DOTA,Tyr3]octreotate in patients with bronchial and gastroenteropancreatic neuroendocrine tumours
Source: Eur J Nucl Med Mol Imaging. 2018 Sep 28;46(3):704–17. doi: 10.1007/s00259-018-4158-1 (PMC6351514; doi:10.1007/s00259-018-4158-1)

**Article title:**

Salvage peptide receptor radionuclide therapy with [^177^Lu-DOTA,Tyr^3^]octreotate in patients with bronchial and gastroenteropancreatic neuroendocrine tumours

**Journal name:**

European Journal of Nuclear Medicine and Molecular Imaging

**Authors:**

van der Zwan W.A.^1^, Brabander T.^1^, Kam B.L.R.^1^, Teunissen J.J.M.^1^, Feelders R.A.^2^, Hofland J.^2^, Krenning E.P.^3^, de Herder W.W.^2^

**Affiliation:**

^1^Department of Radiology & Nuclear Medicine, Erasmus Medical Centre, Rotterdam, The Netherlands

^2^Department of Internal Medicine, Erasmus Medical Centre, Rotterdam, The Netherlands

^3^Cyclotron Rotterdam BV, Erasmus Medical Centre, Rotterdam, The Netherlands

**E-mail address of corresponding author:**

w.vanderzwan@erasmusmc.nl

**Online Resource 1** Correlation PFS of I-PRRT *vs* PFS of R-PRRT with [^177^Lu-DOTA,Tyr^3^]octreotate


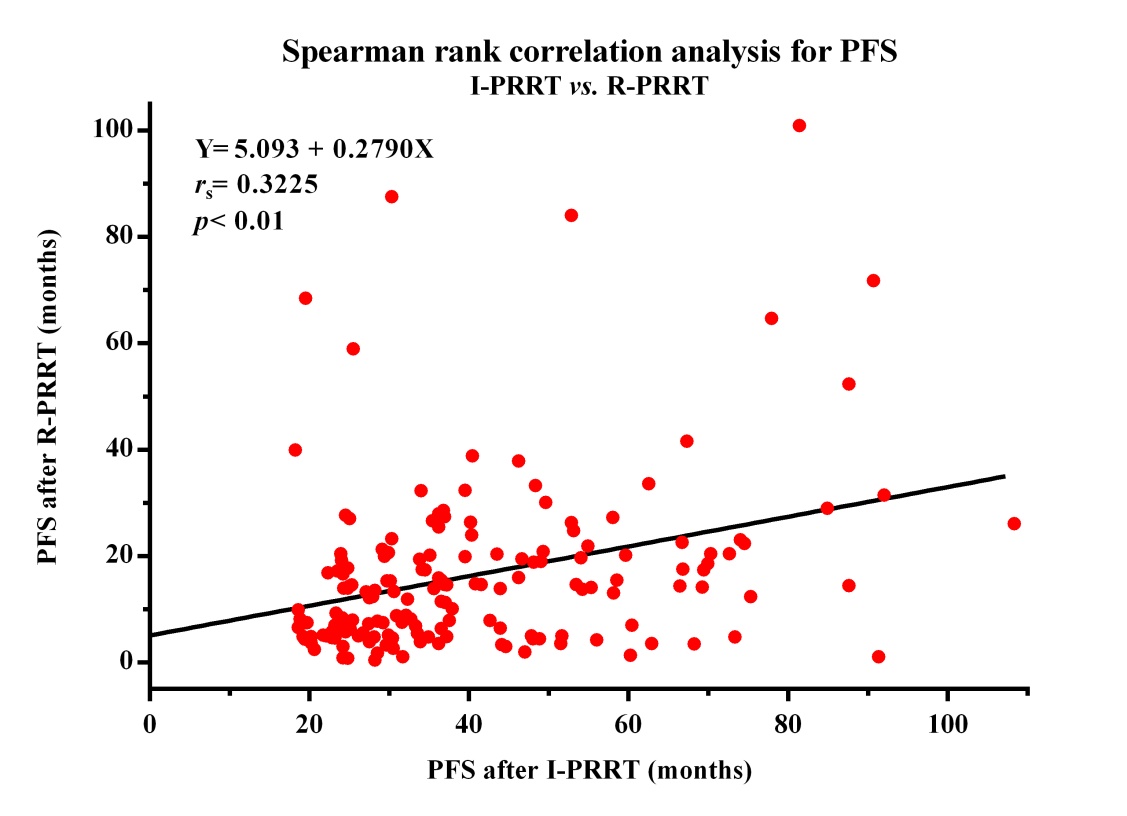

Supplement: Supplementary file 1 — (DOCX 138 kb) [file 259_2018_4158_MOESM1_ESM.docx]
