# Supplementary material for: Salvage peptide receptor radionuclide therapy with [177Lu-DOTA,Tyr3]octreotate in patients with bronchial and gastroenteropancreatic neuroendocrine tumours
Source: Eur J Nucl Med Mol Imaging. 2018 Sep 28;46(3):704–17. doi: 10.1007/s00259-018-4158-1 (PMC6351514; doi:10.1007/s00259-018-4158-1)
Supplement: Supplementary file 2 — (DOCX 31 kb) [file 259_2018_4158_MOESM2_ESM.docx]

**Title:**

Salvage peptide receptor radionuclide therapy with [^177^Lu-DOTA,Tyr^3^]octreotate in patients with bronchial and gastroenteropancreatic neuroendocrine tumours

**Journal name:**

European Journal of Nuclear Medicine and Molecular Imaging

**Authors:**

van der Zwan W.A.^1^, Brabander T.^1^, Kam B.L.R.^1^, Teunissen J.J.M.^1^, Feelders R.A.^2^, Hofland J.^2^, Krenning E.P.^3^, de Herder W.W.^2^

**Affiliation:**

^1^Department of Radiology & Nuclear Medicine, Erasmus Medical Centre, Rotterdam, The Netherlands

^2^Department of Internal Medicine, Erasmus Medical Centre, Rotterdam, The Netherlands

^3^Cyclotron Rotterdam BV, Erasmus Medical Centre, Rotterdam, The Netherlands

**E-mail address of corresponding author:**

w.vanderzwan@erasmusmc.nl

| **Online Resource 2** Krenning score at start I-PRRT and R-PRRT | | | | | |
| --- | --- | --- | --- | --- | --- |
|  | | | | | |
| Grade II | n= | Grade III | n= | Grade IV | n= |
| II II | 1 | III II | 10 | IV II | 3 |
| II III | 7 | III III | 64 | IV III | 20 |
| II IV | 1 | III IV | 19 | IV IV | 41 |
| Baseline uptake on OctreoScan^®^ I-PRRT R-PRRT, not evaluable n= 2 | | | | | |
