# Supplementary material for: Salvage peptide receptor radionuclide therapy with [177Lu-DOTA,Tyr3]octreotate in patients with bronchial and gastroenteropancreatic neuroendocrine tumours
Source: Eur J Nucl Med Mol Imaging. 2018 Sep 28;46(3):704–17. doi: 10.1007/s00259-018-4158-1 (PMC6351514; doi:10.1007/s00259-018-4158-1)
Supplement: Supplementary file 3 — (DOCX 24 kb) [file 259_2018_4158_MOESM3_ESM.docx]

**Article title:**

Salvage peptide receptor radionuclide therapy with [^177^Lu-DOTA,Tyr^3^]octreotate in patients with bronchial and gastroenteropancreatic neuroendocrine tumours

**Journal name:**

European Journal of Nuclear Medicine and Molecular Imaging

**Authors:**

van der Zwan W.A.^1^, Brabander T.^1^, Kam B.L.R.^1^, Teunissen J.J.M.^1^, Feelders R.A.^2^, Hofland J.^2^, Krenning E.P.^3^, de Herder W.W.^2^

**Affiliation:**

^1^Department of Radiology & Nuclear Medicine, Erasmus Medical Centre, Rotterdam, The Netherlands

^2^Department of Internal Medicine, Erasmus Medical Centre, Rotterdam, The Netherlands

^3^Cyclotron Rotterdam BV, Erasmus Medical Centre, Rotterdam, The Netherlands

**E-mail address of corresponding author:**

w.vanderzwan@erasmusmc.nl

| **Online Resource 3** Baseline characteristics of **midgut NETs** in Control group versus Retreatment group prior to any PRRT | | | | | | | | |
| --- | --- | --- | --- | --- | --- | --- | --- | --- |
|  | | | | | | | | |
|  | **Control group – midgut NET (n=63)** | | | **Retreatment group – midgut NET (n=54)** | | | |  |
|  |  |  |  | |  |  |  |  |
|  |  |  |  | |  |  |  |  |
|  | **Yes** | **No** | **Unknown** | | **Yes** | **No** | **Unknown** |  |
| **Baseline Characteristics** | **n (%)** | **n (%)** | **n (%)** | | **n (%)** | **n (%)** | **n (%)** | ***p*-value** |
| Male | 30 (47.6) |  |  | | 35 (64.8) |  |  |  |
| Age^a^ | 63 (37-81) |  |  | | 60 (35-71) |  |  |  |
| Baseline progression^b^ | 27 (42.9) | 14 (22.2) | 22 (34.9) | | 31 (57.4) | 9 (16.7) | 14 (25.9) | 0.14 |
| Prior treatment |  |  |  | |  |  |  |  |
| Surgery | 30 (47.6) | 33 (52.4) | - | | 29 (53.7) | 25 (46.3) | - | 0.58 |
| Chemotherapy | 3 (4.8) | 60 (95.2) | - | | 2 (3.7) | 52 (96.3) | - | 1.00 |
| Radiotherapy | 2 (3.2) | 61 (96.8) | - | | 2 (3.7) | 52 (96.3) | - | 1.00 |
| Somatostatin analogues | 41 (65.1) | 22 (34.9) | - | | 43 (79.6) | 11 (20.4) | - | 0.10 |
| Extent of disease^c^ |  |  |  | |  |  |  |  |
| Limited | 6 (9.5) |  |  | | 8 (14.8) |  |  | 0.40 |
| Moderate | 47 (74.6) |  |  | | 37 (68.5) |  |  | 0.54 |
| Extensive | 10 (15.9) |  |  | | 9 (16.7) |  |  | 1.00 |
| Uptake on OctreoScan^®^ |  |  |  | |  |  |  |  |
| Grade II | 0 (0.0) |  |  | | 3 (5.6) |  |  | 0.10 |
| Grade III | 51 (81.0) |  |  | | 36 (66.7) |  |  | 0.09 |
| Grade IV | 12 (19.0) |  |  | | 15 (27.8) |  |  | 0.28 |
| Liver lesions | 56 (88.9) | 7 (11.1) | - | | 49 (90.7) | 5 (9.3) | - | 0.77 |
| Bone lesions | 10 (15.9) | 53 (84.1) | - | | 13 (24.1) | 41 (75.9) | - | 0.35 |
| Chromogranin A |  |  | 2 (-) | |  |  | 2 (-) |  |
| >2x ULN | 47 (77.0) |  |  | | 36 (69.2) |  |  | 0.40 |
| median (Q_1_-Q_3_)^d^ | 604 (207-2120) |  |  | | 391 (147-1856) |  |  |  |
| Alkaline phosphatase |  |  | 0 (-) | |  |  | 1 (-) |  |
| >2x ULN | 8 (12.7) |  |  | | 6 (11.3) |  |  | 1.00 |
| median (Q_1_-Q_3_)^e^ | 111 (80-158) |  |  | | 108 (74-150) |  |  |  |
| WHO Tumour Grade^f^ |  |  | 48 (-) | |  |  | 33 (-) |  |
| Grade I | 9 (60.0) |  |  | | 6 (28.6) |  |  | 0.78 |
| Grade II | 6 (40.0) |  |  | | 14 (66.7) |  |  | 0.03 |
| Grade III | 0 (0.0) |  |  | | 1 (4.7) |  |  | 0.46 |
| Tumour Response^g^ |  |  | 4 (-) | |  |  | 0 (-) |  |
| CR | 0 (0.0) |  |  | | 0 (0.0) |  |  | - |
| PR | 15 (25.4) |  |  | | 27 (50.0) |  |  | <0.01 |
| SD | 44 (74.6) |  |  | | 27 (50.0) |  |  | 0.01 |
| ^a^Presented as median (range) ^b^Documented progression according RECIST 1.1 ^c^Represents regional distribution of metastatic spread on OctreoScan as described previously [22] | | | | | | | | |
| ^d^Expressed in µg/L ^e^Expressed in U/L ^f^Since 2007 the Ki-67 proliferation index was routinely checked by MIB-1 staining. Patients with an unknown Ki-67 index were mostly treated before 2007 ^g^Tumour response to I-PRRT, evaluation according RECIST 1.1 | | | | | | | | |
