# Supplementary material for: Salvage peptide receptor radionuclide therapy with [177Lu-DOTA,Tyr3]octreotate in patients with bronchial and gastroenteropancreatic neuroendocrine tumours
Source: Eur J Nucl Med Mol Imaging. 2018 Sep 28;46(3):704–17. doi: 10.1007/s00259-018-4158-1 (PMC6351514; doi:10.1007/s00259-018-4158-1)
Supplement: Supplementary file 4 — (DOCX 25 kb) [file 259_2018_4158_MOESM4_ESM.docx]

**Article title:**

Salvage peptide receptor radionuclide therapy with [^177^Lu-DOTA,Tyr^3^]octreotate in patients with bronchial and gastroenteropancreatic neuroendocrine tumours

**Journal name:**

European Journal of Nuclear Medicine and Molecular Imaging

**Authors:**

van der Zwan W.A.^1^, Brabander T.^1^, Kam B.L.R.^1^, Teunissen J.J.M.^1^, Feelders R.A.^2^, Hofland J.^2^, Krenning E.P.^3^, de Herder W.W.^2^

**Affiliation:**

^1^Department of Radiology & Nuclear Medicine, Erasmus Medical Centre, Rotterdam, The Netherlands

^2^Department of Internal Medicine, Erasmus Medical Centre, Rotterdam, The Netherlands

^3^Cyclotron Rotterdam BV, Erasmus Medical Centre, Rotterdam, The Netherlands

**E-mail address of corresponding author:**

w.vanderzwan@erasmusmc.nl

| **Online Resource 4** Baseline characteristics of **pancreatic NETs** in Control group versus Retreatment group prior to any PRRT | | | | | | | | |
| --- | --- | --- | --- | --- | --- | --- | --- | --- |
|  | | | | | | | | |
|  | **Control group – pancreatic NET (n=20)** | | | **Retreatment group – pancreatic NET (n=53)** | | | |  |
|  |  |  |  | |  |  |  |  |
|  |  |  |  | |  |  |  |  |
|  | **Yes** | **No** | **Unknown** | | **Yes** | **No** | **Unknown** |  |
| **Baseline Characteristics** | **n (%)** | **n (%)** | **n (%)** | | **n (%)** | **n (%)** | **n (%)** | ***p*-value** |
| Male | 8 (40.0) |  |  | | 24 (45.3) |  |  |  |
| Age^a^ | 62 (38-81) |  |  | | 54 (32-78) |  |  |  |
| Baseline progression^b^ | 11 (55.0) | 4 (20.0) | 5 (25.0) | | 25 (47.2) | 7 (13.2) | 21 (39.6) | 0.61 |
| Prior treatment |  |  |  | |  |  |  |  |
| Surgery | 8 (40.0) | 12 (60.0) | - | | 20 (37.7) | 33 (62.3) | - | 1.00 |
| Chemotherapy | 1 (5.0) | 19 (95.0) | - | | 3 (5.7) | 50 (94.3) | - | 1.00 |
| Radiotherapy | 0 (0) | 20 (100) | - | | 3 (5.7) | 50 (94.3) | - | 0.56 |
| Somatostatin analogues | 7 (35.0) | 13 (65.0) | - | | 17 (32.1) | 36 (67.9) | - | 1.00 |
| Extent of disease^c^ |  |  |  | |  |  |  |  |
| Limited | 1 (5.0) |  |  | | 7 (13.2) |  |  | 0.43 |
| Moderate | 16 (80.0) |  |  | | 44 (83.0) |  |  | 0.74 |
| Extensive | 3 (15.0) |  |  | | 2 (3.8) |  |  | 0.12 |
| Uptake on OctreoScan^®^ |  |  |  | |  |  |  |  |
| Grade II | 0 (0.0) |  |  | | 2 (3.8) |  |  | 1.00 |
| Grade III | 13 (65.0) |  |  | | 25 (47.2) |  |  | 0.20 |
| Grade IV | 7 (35.0) |  |  | | 26 (49.1) |  |  | 0.10 |
| Liver lesions | 14 (70.0) | 6 (30.0) | - | | 46 (86.8) | 7 (13.2) | - | 0.17 |
| Bone lesions | 0 (0.0) | 20 (100.0) | - | | 9 (17.0) | 44 (83.0) | - | 0.06 |
| Chromogranin A |  |  | 1 (-) | |  |  | 3 (-) |  |
| >2x ULN | 11 (57.9) |  |  | | 33 (66.0) |  |  | 0.58 |
| median (Q_1_-Q_3_)^d^ | 212 (107-516) |  |  | | 393 (150-1155) |  |  |  |
| Alkaline phosphatase |  |  | 0 (-) | |  |  | 2 (-) |  |
| >2x ULN | 4 (20.0) |  |  | | 9 (17.6) |  |  | 0.74 |
| median (Q_1_-Q_3_)^e^ | 118 (71-172) |  |  | | 108 (77-171) |  |  |  |
| WHO Tumour Grade^f^ |  |  | 12 (-) | |  |  | 19 (-) |  |
| Grade I | 2 (25.0) |  |  | | 7 (20.6) |  |  | 1.00 |
| Grade II | 5 (62.5) |  |  | | 24 (70.6) |  |  | 0.03 |
| Grade III | 1 (12.5) |  |  | | 3 (8.8) |  |  | 1.00 |
| Tumour Response^g^ |  |  | 0 (-) | |  |  | 1 (-) |  |
| CR | 0 (0) |  |  | | 1 (1.9) |  |  | 1.00 |
| PR | 14 (70.0) |  |  | | 36 (69.2) |  |  | 1.00 |
| SD | 6 (30.0) |  |  | | 15 (28.9) |  |  | 1.00 |
| ^a^Presented as median (range) ^b^Documented progression according RECIST 1.1 ^c^Represents regional distribution of metastatic spread on OctreoScan as described previously [22] | | | | | | | | |
| ^d^Expressed in µg/L ^e^Expressed in U/L ^f^Since 2007 the Ki-67 proliferation index was routinely checked by MIB-1 staining. Patients with an unknown Ki-67 index were mostly treated before 2007 ^g^Tumour response to I-PRRT, evaluation according RECIST 1.1 | | | | | | | | |
